# Low skeletal muscle mass and post-operative complications after surgery for liver malignancies: a meta-analysis

**DOI:** 10.1007/s00423-022-02541-5

**Published:** 2022-05-18

**Authors:** Maximilian Thormann, Jazan Omari, Maciej Pech, Robert Damm, Roland Croner, Aristotelis Perrakis, Alexandra Strobel, Andreas Wienke, Alexey Surov

**Affiliations:** 1 Clinic for Radiology and Nuclear Medicine, University Hospital Magdeburg, Leipziger Str. 44, 39120 Magdeburg, Germany; 2 Department of General-, Visceral-, Vascular- and Transplantation Surgery, University Hospital Magdeburg, Magdeburg, Germany; 3 Institute of Medical Epidemiology, Biometry, and Informatics, Martin Luther University, Halle-Wittenberg, Germany; 4 Profile Area Clinical Studies & Biostatistics, Institute of Medical Epidemiology, Biostatistics, and Informatics, Martin-Luther-University, Halle-Wittenberg Halle, Germany

**Keywords:** Computed tomography assessed sarcopenia, Post-operative complications, Hepatic malignancies, Meta-analysis

## Abstract

**Purpose:**

To assess the influence of low skeletal muscle mass (LSMM) on post-operative complications in patients with hepatic malignancies grade (Clavien Dindo ≥ 3) undergoing resection.

**Methods:**

MEDLINE, Cochrane, and SCOPUS databases were screened for associations between sarcopenia and major post-operative complications (≥ grade 3 according to Clavien-Dindo classification) after resection of different malignant liver tumors. RevMan 5.3 software was used to perform the meta-analysis. The methodological quality of the included studies was assessed according to the QUIPS instrument.

**Results:**

The analysis included 17 studies comprising 3157 patients. Subgroup analyses were performed for cholangiocarcinoma (CCC), colorectal cancer (CRC) liver metastases, and hepatocellular carcinoma (HCC). LSMM as identified on CT was present in 1260 patients (39.9%). Analysis of the overall sample showed that LSMM was associated with higher post-operative complications grade Clavien Dindo ≥ 3 (*OR* 1.56, 95% *CI* 1.25–1.95, *p* < 0.001). In the subgroup analysis, LSMM was associated with post-operative complications in CRC metastases (*OR* 1.60, 95% *CI* 1.11–2.32, *p* = 0.01). In HCC and CCC sub-analyses, LSMM was not associated with post-operative complications in simple regression analysis.

**Conclusion:**

LSMM is associated with major post-operative complications in patients undergoing surgery for hepatic metastases and it does not influence major post-operative complications in patients with HCC and CCC.

## Introduction

Sarcopenia has been found to be an indicator of poor prognosis in oncologic diseases. It is defined as the loss of or low muscle mass, low muscle strength, and impaired muscle quality [1]. Commonly used indicators for sarcopenia are low skeletal muscle mass (LSMM) and muscle density, both of which can be assessed on computed tomography (CT) scans [2]. LSMM has been associated with poorer survival in malignancies such as gastric and esophageal cancer, colorectal cancer, pancreatic cancer, and lymphoma, among others [3–7]. It has also been associated with dose-limiting toxicity (DLT) and with higher rates of cardiac and pulmonary complications in oncologic patients [8, 9]. For post-operative outcomes, a negative influence for post-operative LSMM has been shown for extrahepatic cancer entities [10–12].

The influence of LSMM on post-operative complications for cancer patients has been shown in meta-analyses for gastric cancer (*OR* 2.17, 95% *CI* 1.53–3.08) [13] and colorectal cancer (*OR* = 1.82, 95% *CI* = 0.36–2.44) [14]. In esophageal cancer, pre-operative LSMM was associated with higher rates of post-operative pulmonary complications (*OR* 2.03, 95% *CI* 1.32–3.119), but not with higher rates of complications as defined by Clavien Dindo (*OR* 1.19, 95% *CI* 0.78 to 1.81) [15, 16]. No association with overall post-operative complications was found in a meta-analysis for pancreatic cancer (*OR* 0.96, 95% *CI* 0.78–1.19), yet sarcopenic patients showed higher peri-operative mortality (*OR* 2.40, 95% *CI* 1.19–4.85) [17].

The impact of LSMM on patient outcome after surgery for hepatic malignancies is not yet clear. For most primary and secondary liver tumors, surgical resection is the cornerstone of curative treatment approaches. However, liver resections remain major surgical procedures and are still associated with relevant post-operative morbidity and mortality and careful patient selection remains crucial in order to improve patient outcome [18, 19]. Prognostic indicators for patient outcome after liver surgery are wanted. It is known that age, performance status, comorbidities, and lymph node status, among others, influence post-operative complications and outcome [20, 21]. However, discriminatory accuracy of prognostic scores has been limited [22].

The aim of this study is to systematically assess the influence of LSMM on patient post-operative outcomes (grade Clavien Dindo ≥ 3) after hepatic resection for primary and secondary liver malignancies.

## Methods

### Search strategy

**Fig. 1:**
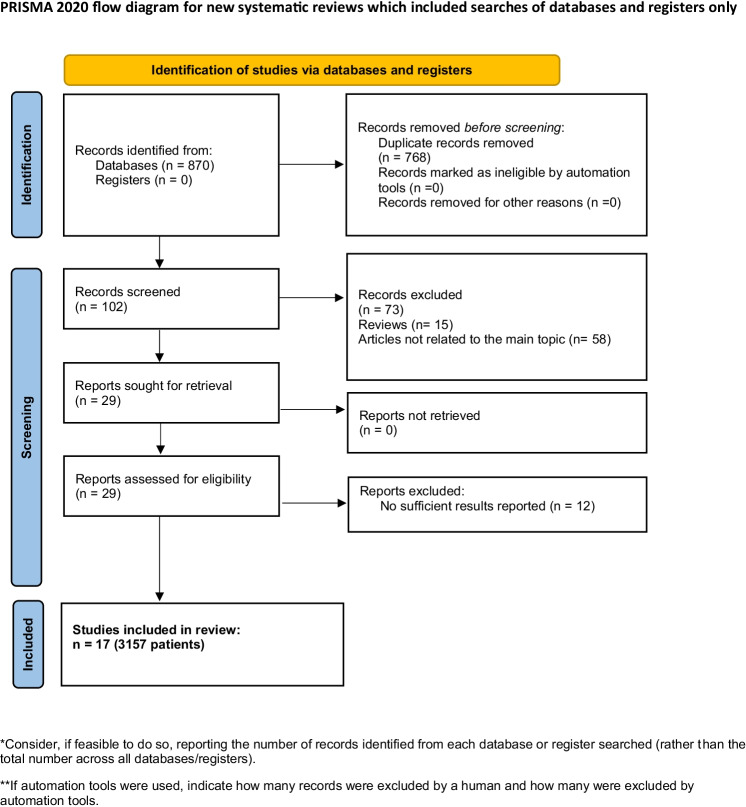
Prisma flow chart of data acquisition

For the present analysis, we performed a search within MEDLINE library, Cochrane, SCOPUS, and Web of Science data bases using the Preferred Reporting Items for Systematic Reviews and Meta-Analyses statement (PRISMA) (Fig. 1) [23]. The search was performed according to the recommendation for literature search in surgical systematic reviews [24]. Occurrence of major (≥ grade 3 according to Clavien-Dindo classification) postoperative complications after resection of different malignant liver tumors was the endpoint of the present meta-analysis.

The following search criteria were used: “sarcopenia OR low skeletal muscle mass OR body composition OR skeletal muscle index AND liver AND postoperative complications OR postoperative complication.” The last search was performed in March 2022. Inclusion criteria for the articles were as follows:

- original investigations with humans;
- patients with malignant hepatic tumors treated by surgical resection;
- estimation of presurgical LSMM/sarcopenia;
- reported data about influence of LSMM/sarcopenia on occurrence of postoperative complications (odds ratios and 95% *CI*’s).

Exclusion criteria were as follows:

- review articles, case reports, and letters;
- non-English language;
- experimental studies;
- missing of statistical data regarding influence of LSMM/sarcopenia on occurrence of postoperative complications (odds ratios and 95% *CI*’s).

### Data extraction

At first, the abstracts were checked. Duplicate articles, review articles, experimental studies, case reports, and non-English publications were excluded. Furthermore, the full texts of the remaining articles were analyzed. Studies with no sufficient data were excluded. The remaining articles met the inclusion criteria. The following data were acquired for the analysis: authors, year of publication, type of tumors, number of patients, prevalence of LSMM/sarcopenia, and statistical data about influence of LSMM/sarcopenia on outcomes (odds ratios and 95% *CI*’s).

### Meta-analysis

Three observers (AS, MT and AW) in consensus analyzed the methodological quality of the included 17 studies according to the Quality in Prognosis Studies Instrument (QUIPS) instrument [25]. Risk of bias of studies was considered low if ≤ 2 items were rated “low risk” or “moderate risk.” Risk of bias was considered high if ≥ 1 item was rated “high risk.” Furthermore, a funnel plot was constructed to analyze a possible publication bias and asymmetry was quantified by using the Egger test [26]. *p* value of less than 0.05 indicated publication bias.

The RevMan 5.3 (Computer program, version 5.3. Copenhagen: The Nordic Cochrane Center, the Cochrane Collaboration, 2014) was used [27, 28]. Heterogeneity was calculated by means of the index I^2^. DerSimonian and Laird random-effects models with inverse-variance weights were performed [29].

## Results

### Description of included studies

According to the search strategy, 870 records were initially identified. After exclusion of duplicate records, 102 studies were screened. Records that did not meet the inclusion criteria (*n* = 73), reviews, and those not related to the topic under investigation were excluded. Of the remaining 29 studies, 12 did not report sufficient results. Ultimately, 17 studies with 3157 patients were included in our analysis (Fig. 1). Of these, five studies were from Asia (4 from Japan, one from China), ten from Europe (three from Germany, two from France, one from Austria, one from Sweden, one from the Netherlands, one from Italy, one from Switzerland), and two were from the USA. Included studies were published between 2011 and 2022. All studies were retrospective in nature.

The assessed liver malignancies were as follows: cholangiocarcinoma (CCC) (2 studies, 176 patients), colorectal liver metastases (6 studies, 1188 patients), HCC (6 studies, 1108 patients), and different primary and secondary hepatic malignancies (3 studies, 685 patients). Study characteristics are summarized in Table 1. Altogether, there were 2058 men (64.4%) and 1139 women (35.6%) included in the studies. The Egger test did not identify a publication bias among the included articles (*p* = 0.10).

**Table 1:** Characteristics of included studies and definitions of sarcopenia

Author	Year	Country	Entity	Sample size	Study design	LSMM assessment	Sex-specific cut-off values	Definition of cut-off values
Okumura et al. [68]	2017	Japan	CCC	109	Retrospective	SMI	Males: 52.5 cm^2^/m^2^; females: 41.2 cm^2^/m^2^	Receiver operator characteristics curve
Zhou et al. [50]	2015	China	CCC	67	Retrospective	SMI	Males: 43.75 cm^2^/m^2^; females: 41.10 cm^2^/m^2^	Vledder et al. 2012 [69]
Bajric et al. [70]	2022	Austria	CRC	315	Retrospective	SMI	Males: 59.1 cm^2^/m^2^; females 48.4 cm^2^ /m^2^	Prado et al. 2008 [71]
Eriksson et al. [72]	2017	Sweden	CRC	225	Retrospective	SMI	Males: 52.4 cm^2^/m^2^; females 38.5 cm^2^/m^2^	Prado et al. 2008 [71]
Kobayashi et al. [73]	2018	Japan	CRC	124	Retrospective	SMI	Males: 40.31 cm^2^/m^2^; females: 30.88 cm^2^/m^2^	Hamaguchi et al. 2017 [74]
Lodewick et al. [41]	2015	Netherlands	CRC	171	Retrospective	SMI	Males: 43 cm^2^ /m^2^ (BMI < 25); 53 cm^2^/m^2^ (BMI > 25); females: 41 cm^2^/m^2^	Martin et al. 2013 [75]
Peng et al. [31]	2011	USA	CRC	259	Retrospective	TPA	500 mm^2^/m^2^	Optimum stratification
Runkel et al. [52]	2021	Germany	CRC	94	Retrospective	SMI	Males: 52.4 cm^2^/m^2^; females 38.5 cm^2^/m^2^	Prado et al. 2008 [71]
Kobayashi et al. [73]	2019	Japan	HCC	465	Retrospective	SMI	Males: 40.31 cm^2^/m^2^; females: 30.88 cm^2^/m^2^	Hamaguchi et al. 2017 [74]
Kroh et al. [51]	2019	Germany	HCC	70	Retrospective	SMI	Males: 43 cm^2^/m^2^ (BMI < 25); 53 cm^2^/m^2^ (BMI > 25); females: 41 cm^2^/m^2^	Martin et al. 2013 [75]
Meister et al. [76]	2022	Germany	HCC	100	Retrospective	SMI	Males: 50.0 cm^2^/m^2^; females 39.0 cm^2^/m^2^	Eslamparast et al. 2018 [77]
Seror et al. [53]	2021	France	HCC	110	Retrospective	SMI	Males: 52 cm^2^/m^2^; females 38 cm^2^/m^2^	Prado et al. 2008 [71]
Takagi et al. [78]	2016	Japan	HCC	254	Retrospective	SMI	Males: 46.4 cm^2^/m^2^; females: 37.6 cm^2^/m^2^	Optimum stratification
Voron et al. [79]	2015	France	HCC	109	Retrospective	SMI	Males: 52.4 cm^2^/m^2^; females: 38.9 cm^2^/m^2^	Prado et al. 2008 [71]
Valero et al. [32]	2015	USA	HCC, CCC	96	Retrospective	TPA/TPV	TPA: Males: 642.1 mm^2^/m^2^; females 784.0 mm^2^/m^2^ TPV: Males 34.14 cm^3^/m; females 22.93 cm^3^/m	Peng et al. 2011 [31] Prado et al. 2008 [71]
Berardi et al. [30]	2020	Italy	Hepatic malignancies	234	Retrospective	SMI / handgrip strength	SMI: Males 53.5 cm^2^/m^2^; females 40.8 cm^2^/m^2^ Handgrip: Males 30 kg; females 20 kg	SMI: receiver operating characteristic curve analysis
Martin et al. [49]	2022	Switzerland	Liver malignancies, infections	355	Retrospective	SMI	Males: 52.4 cm^2^/m^2^; females: 38.9 cm^2^/m^2^	Prado et al. 2008 [71]

**Fig. 2:**
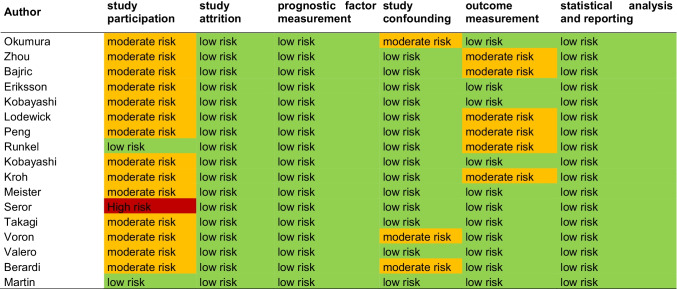
QUIPS assessment of included studies

According to the QUIPS checklist, 16/17 (94.1%) studies had an overall low risk of bias. A high risk of bias was assigned to one study due to a possible bias in patient selection criteria (Fig. 2). No studies were excluded due to a risk of bias.

### Assessment of LSMM

Most studies used the skeletal muscle index (SMI) at the level of the third lumbar vertebra to measure LSMM (14 studies, 82.4%). One study (5.9%) used a combination of SMI and muscle strength to define LSMM [30]. The total psoas area (TPA) and the total psoas volume (TPV) were applied in one study, respectively [31, 32].

LSMM as identified by pre-operative CT scans was identified in 1260 patients (39.9%). In the subgroup analysis, the rate of sarcopenic patients ranged from 31.8% in the HCC cohort to 58.0% in the CCC cohort.

### Meta-analysis for major post-operative complications

**Fig. 3:**
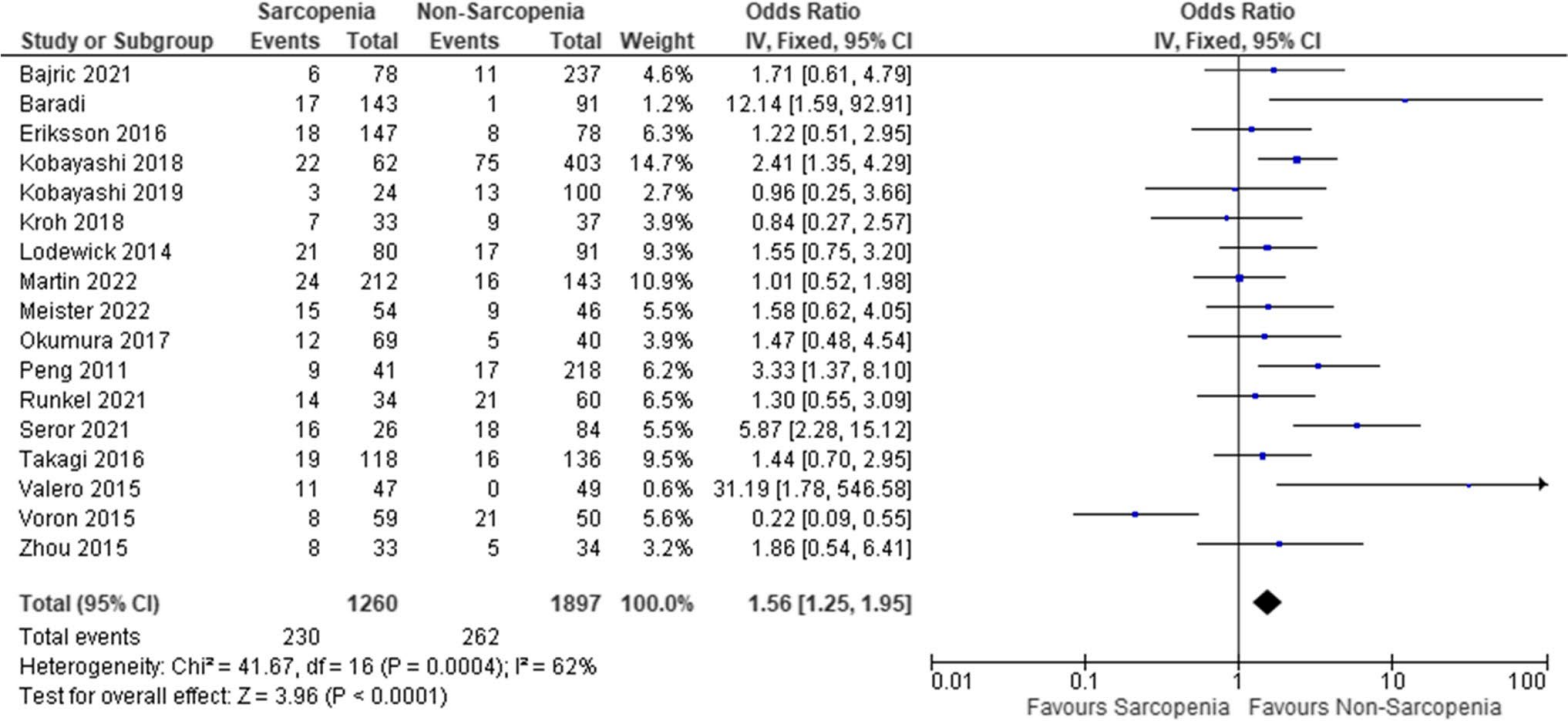
Forest plots comparing major post-operative complications in sarcopenia versus non-sarcopenic patients with hepatic malignancies

Regression of the aggregated data showed that across all studies, LSMM was associated with higher major post-operative complications (*OR* 1.56, 95% *CI* 1.25–1.95, *p* < 0.001). The studies showed a high heterogeneity (*I*^2^ = 62%) (Fig. 3).

In the subgroup analyses, the influence of LSMM for the different tumor entities was analyzed.

**Fig. 4:**
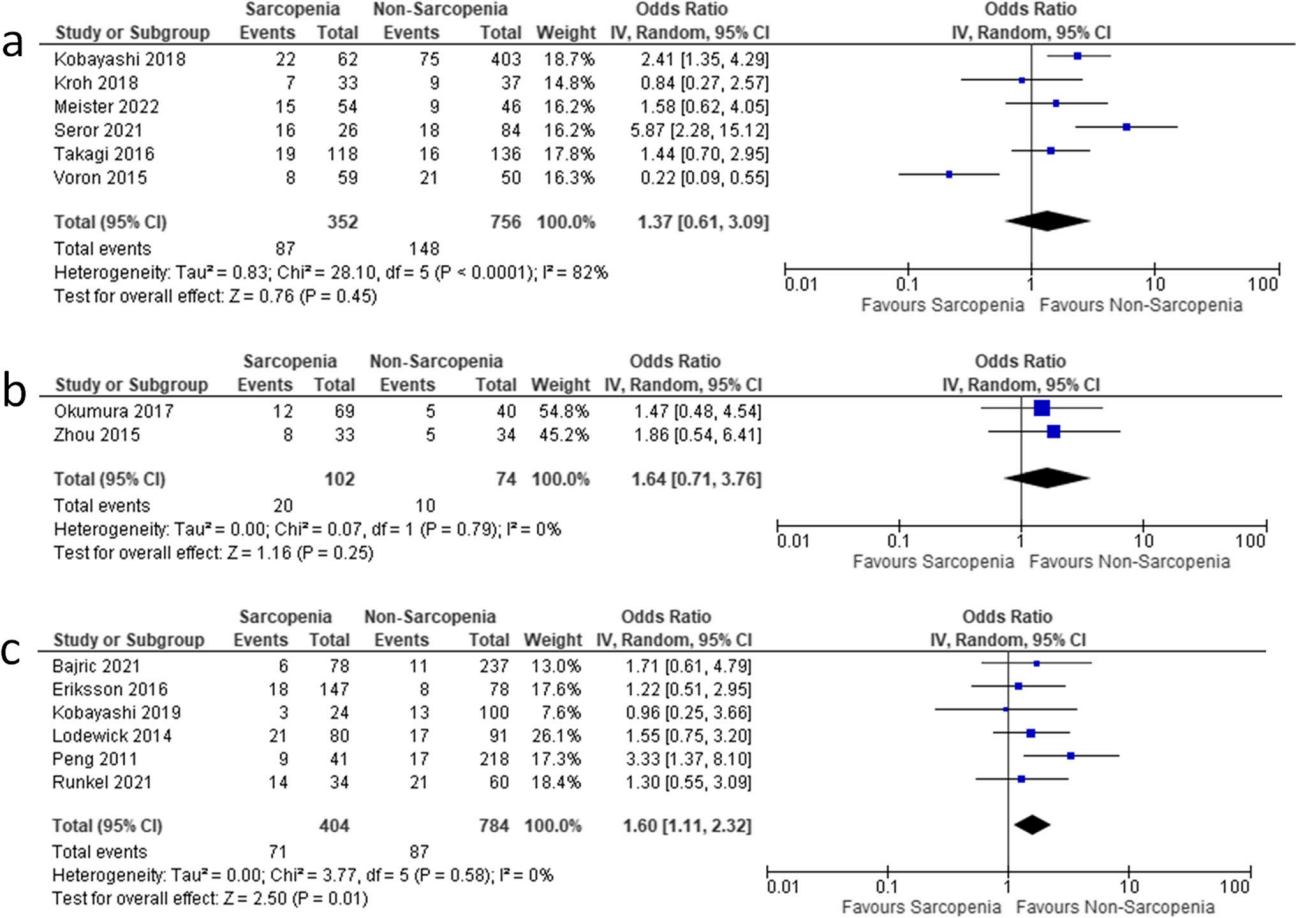
Associations between low skeletal muscle mass (LSMM) and major post-operative complications for HCC (**a**), CCC (**b**), and colorectal liver metastases (**c**)

In CCC, simple regression did not show an association between LSMM and major complications (*OR* 1.64, 95% *CI* 0.71–3.76, *p* = 0.25). There was no heterogeneity between the studies (*I*^2^ = 0%) (Fig. 4b).

In HCC, an association between pre-operative LSMM and major post-operative complications was as follows: *OR* 1.37, 95% *CI* 0.61–3.09, *p* = 0.45. There was high heterogeneity between the studies (*I*^2^ = 82%) (Fig. 4a).

In colorectal liver metastases, simple regression showed that LSMM was associated with higher post-operative complications (*OR* 1.60, 95% *CI* 1.11–2.32, *p* = 0.01). There was no heterogeneity between the studies (*I*^2^ = 0%) (Fig. 4c).

## Discussion

To the best of our knowledge, this is the first meta-analysis assessing the impact of LSMM on major post-operative complications after hepatic resection for various hepatic malignancies in a large sample. It is shown that pre-operative LSMM is associated with higher rates of major complications in patients with hepatic malignancies undergoing hepatic resection.

The importance of LSMM for oncologic patients has been underlined for various clinical features. It has been shown that LSMM is associated with higher rates of post-operative cardiac and pulmonary complications in gastric cancer patients [9]. In non-small-cell lung cancer, patients with LSMM undergoing surgery had a lower 5-year OS and a lower disease-free survival rate [33]. An association between LSMM and dose limiting toxicity in oncologic patients has also been identified [8]. Metabolism of anti-cancer drugs may also be affected [34, 35]. It has been reported that LSMM is associated with elevated intracellular inflammation, oxidative stress, and high protein consumption [1, 36].

Previous meta-analyses have found an association of LSMM with OS after local therapy for CRC liver metastases [20]. For instance, Levolger et al. found poorer OS in patients with LSMM undergoing surgery for gastrointestinal hepatopancreatobiliary malignancies [37]. Trejo-Avila et al. reported an association between LSMM and worse post-operative OS in patients with CRC [38]. Xu et al. found shorter post-operative OS in HCC patients undergoing hepatectomy [39]. Simonsen et al. identified LSMM as an increased risk for post-operative complications after surgery for gastrointestinal cancer [40]. However, a sub-analysis did not find an association between LSMM and post-operative complications after surgery for CRC liver metastases (*RR* 1.91, 95% *CI* 0.97–3.75). The fact that their analysis included only two studies [31, 41] and a low number of patients may explain the different results compared to our analysis. Also, no significant association was found for liver cancer (*RR* 1.25, 95% *CI* 0.92–1.71).

With regard to complications after surgery, an association between LSMM and post-operative outcomes according to the Clavien Dindo score was reported for cancer patients after gastrectomy (*OR* 2.17, 95% *CI* 1.53–3.08) [13] and surgery for colorectal cancer (*OR* = 1.82, 95% *CI* = 1.36–2.44) [14]. Single studies have found LSMM to be associated with post-operative complications for pancreatic cancer [42], yet a meta-analysis did not find a significant association (*OR* 0.96, 95% *CI* 0.78–1.19) [17]. However, patients with LSMM showed a higher peri-operative mortality after pancreatic surgery (*OR* 2.40, 95% *CI* 1.19–4.85) [17]. Similarly, in esophageal cancer, no significant influence of LSMM on post-operative complications as defined by the Clavien Dindo grading was found (*OR* 1.19, 95% *CI* 0.78 to 1.81), yet patients with LSMM exhibited higher rates of respiratory complications [15, 16]. It was also found that patients with LSMM had higher rates of nosocomial infections after colectomy [43]. In HCC, one study found higher rates of post-operative complications in patients having undergone either hepatic resection or radiofrequency ablation (RFA) [12, 44]. In patients undergoing liver resection, patients with LSMM exhibited smaller preoperative total functional liver volume [45, 46]. Cao et al. reported that different measures of LSMM, such as SMI and PMI, can predict major post-operative complications following surgery for hepatopancreatobiliary malignancies [47]. In a meta-analysis by Zhang et al. including patients undergoing treatment for primary hepatic malignancies, the rate of major complications according to Clavien Dindo ≥ 3 was not associated with the presence of LSMM [48].

In the present study, the association of LSMM with major post-operative complications was significant, yet discrete, in the aggregate analysis. A significant association was found only for CRC liver metastases in the subgroup analysis. In HCC and CCC sub-analyses, no significant impact of sarcopenia was found. The impact of sarcopenia did not differ significantly between primary hepatic malignancies and metastases. Of the studies combining different malignancies [30, 32, 49], two did not refer to SMI as a measure of sarcopenia alone, but Berardi et al. used the combination of reduced muscle mass and strength as a definition of LSMM, while Valero et al. applied TPA and TPV. Our overall results exhibit a somewhat lower OR for post-operative complications for liver resection than those published for gastrectomy or surgery for CRC [13, 14].

In our analysis, LSMM was only associated with major post-operative complications in patients with CRC liver metastases. The reason for this remains unknown. We hypothesize that the number of lesions resected is higher in liver metastases than in primary liver tumors. However, the number of lesions resected is often not indicated in the studies so that a detailed analysis is not possible.

Of the included 17 studies in our analysis, only seven studies detailed the kind of complications that occurred after surgery. The most common surgical complications were intraabdominal abscesses and biliary leakages [32, 41, 50–52]. Peng et al. reported post-operative bleeding as the most frequent surgical complication, while ascites was the most common complication in Berardi et al. [30, 31]. Among non-surgical complications, respiratory complications including pneumonia and cardiovascular complications were most frequently mentioned [41, 50, 51, 53]. Pleural effusion requiring drainage was the most common cited complication in the study by Peng et al. [31]. Due the low number of studies giving details on the category of complications, no sub-analysis was performed.

The present study adds to the evidence that LSMM is associated with major post-operative complications in cancer patients. The novelty of the present work is that for the first time a selective analysis of post-operative complications after surgery for different liver tumors was performed. This sub-analysis for different entities was not performed in other meta-analyses. The fractions of patients with LSMM in our studies are within the ranges reported for cancer patients in the literature [12, 54, 55]. The prevalence of LSMM differed depending on tumor entity, with HCC showing the lowest rate (29.6%) and CCC the highest rate (58.0%). It must be noted, however, that heterogeneity in the overall sample was relatively high at 62%. This is due to the low number of studies involved and heterogeneous tumor entities. This may affect the generalization of our results. Nevertheless, our findings implicate importance for the daily clinical practice.

Measurements of LSMM are easy to acquire as most patients undergo routine staging CT scans prior to surgery. Unlike other factors influencing survival, LSMM is a modifiable factor. Early identification is important and may induce treatment and improve patient outcomes. The literature has shown that the vulnerability of patients with LSMM/sarcopenia stems from limited mobility as well as from distorted metabolic and physiological pathways. Patients with LSMM have activated systemic inflammatory pathways and might have increased metabolic activity, leading to inflammation and muscle wasting [56]. Skeletal muscle has both endocrine and paracrine functions that may be inhibited by LSMM [57]. Some of these myokines may possess anti-neoplastic effect and suppress tumorigenesis [58, 59]. Thus, multimodal interventions, including supervised physical exercise and improving nutritional status, may potentially inhibit cancer cell division in patients with LSMM and improve quality of life [60]. There is increasing evidence that physical training in oncologic patients can improve muscle function [61, 62]. At the same time, dietary supplements and high protein diet may prevent the loss of muscle mass [63].Yamamoto et al. have shown that pre-operative exercise and nutritional supplementation may reduce sarcopenia and improve post-operative outcomes in patients undergoing surgery for gastric cancer [62]. For head and neck squamous cell cancer (HNSCC), Kabarriti et al. reported improved survival (non-significant) and reduced disease progression following an additive nutritional program [64]. The two may also be intertwined, as Yokoyama et al. reported a correlation between physical activity and nutritional status before hepato-pancreato-biliary surgery [65]. Also, Kelly et al. have shown that post-operative complications represent a major risk factor for hospital readmissions after gastrointestinal surgery [66]. This can potentially be reduced by preventive interventions. Physical exercise and nutritional supplementation should be started early in the disease process and intensified prior to surgery to potentially improve outcomes. Further research will be necessary to determine the best training and nutritional protocol for patients with LSMM before surgery.

Our analysis has several limitations that need to be addressed. All studies included were retrospective. Some suffered from selection bias. We included studies in English language only. While the total number of studies screened was high, subgroup analyses may suffer from the low number of studies available. The articles included tumors of different entities and at different stages with varying surgical procedures, being reflected by the high heterogeneity of the studies in the aggregate analysis. Furthermore, definitions and measurements of LSMM were different among included studies (Table 1). Most studies in our analysis measured LSMM by means of CT, while one study used a combination of CT and handgrip strength. While CT is considered the gold standard, correlation between different methods of assessing sarcopenia is low, as was recently reported in a study by Simonsen et al. [67].

In conclusion, our meta-analysis showed that LSMM is a discrete but significant factor of post-operative complications in patients undergoing surgery for colorectal liver metastases. The presence of LSMM should be specifically mentioned in medical reports in patients with colorectal liver metastases. Addressing sarcopenia could potentially improve outcome in this patient group. LSMM does not influence major post-operative complications in patients with HCC and CCC.

## Abbreviations

CCC: Cholangiocarcinoma
CRC: Colorectal carcinoma
CT: Computed tomography
DLT: Dose limiting toxicity
HCC: Hepatocellular carcinoma
LSMM: Low skeletal muscle mass
PMI: Psoas muscle index
QUIPS: Quality in prognosis studies instrument
SMI: Skeletal muscle index
TPA: Total psoas area
TPV: Total psoas volume
